# A bibliometric and visualized analysis of research on mitochondria in myocardial ischemia from 2015 to 2024

**DOI:** 10.3389/fcvm.2025.1547604

**Published:** 2025-07-07

**Authors:** Yang Lu, Fanghe Li, Qiong Wu, Qingteng Zhai, Xinyi Li, Weibin Xie, Shuwen Guo, Kuo Gao

**Affiliations:** ^1^ School of Traditional Chinese Medicine, Beijing University of Chinese Medicine, Beijing, China; ^2^ Institute of Basic Theory for Chinese Medicine, China Academy of Chinese Medical Sciences, Beijing, China; ^3^ Fangshan Hospital, Beijing University of Chinese Medicine, Beijing, China

**Keywords:** bibliometric analysis, myocardial ischemia, mitochondria, hotspots, visualization, VOSviewer, CiteSpace

## Abstract

**Background:**

Mitochondria, as the powerhouse of myocardial energy metabolism, have garnered considerable interest in the realm of myocardial ischemia (MI) therapy research. The purpose of this study is to illustrate the hot spots and frontiers of mitochondrial research in MI in the past decade through bibliometric analysis.

**Methods:**

Both articles and reviews of mitochondrial research in MI from 2015 to 2024 were retrieved from the Web of Science Core Collection database. Bibliometric analysis was performed mainly using CiteSpace and VOSviewer.

**Results:**

The analysis encompassed 4,387 papers. The annual publications exhibited a significant increasing trend, rising from 342 publications in 2015 to 541 publications in 2024. China, the United States of America, and Germany emerged as the top three contributors to MI related mitochondria studies. The Air Force Medical University, China, was the leading publisher. Chattipakorn, Nipon (Chiang Mai University), Chattipakorn, Siriporn C (Chiang Mai University), Zhou, Hao (Zhejiang University) were the top three most active and influential scholars based on the H-index. *International Journal of Molecular Sciences* was the most co-cited journal in this field. Until 2024, the keywords with the strongest citation bursts included mitophagy, transplantation, homeostasis, and myocardial ischemia-reperfusion. The current research trend is to translate mitochondria-related diagnostic and therapeutic drugs for MI into the clinic.

**Conclusions:**

Exploring MI from a mitochondrial perspective has great potential. By revealing the knowledge structure of this field, this study helps to provide scholars in related fields with a comprehensive understanding of the field and develop collaborations.

## 1 Introduction

Ischemic heart disease (IHD), especially myocardial infarction, is the leading cause of death worldwide. Myocardial infarction originates from a reduction or cessation of coronary blood flow, leading to the death of myocardial cells and the formation of irreversible fibrotic scars (1, 2). Recently, numerous cellular processes have been implicated in myocardial ischemia (MI), including mitochondrial metabolism, oxygen provision, and hypoxia-induced signal transduction (3). Mitochondria, pivotal cellular organelles that modulate cell shape, distribution, and function, are increasingly recognized as key players in mechanisms of MI (4, 5). The heart's energy and metabolic demands are multifaceted and highly dynamic (6, 7). In recent years, many studies confirmed that mitochondrial metabolism and quality control serve as pharmacological targets in cardiovascular diseases, especially MI (8, 9). Studies have shown that in the case of MI, the application of drug therapy to change mitochondrial function provides important advantages for the regulation of cardiac metabolism. For example, paying attention to the morphology and mechanism integrity of mitochondria (10), exploring the effects of drugs and treatments on mitophagy, and maintaining the relationship between mitochondrial function and myocardial protection are all current research issues in ischemic cardiomyopathy around mitochondria (11). Consequently, mitochondria have emerged as a focal point in MI research, offering valuable insights into the pathogenesis, biomarkers, and targeted therapies for ischemic heart disease (12).

Bibliometrics, a widely employed research method, facilitates the analysis of developmental trends within a specific field. This method provides researchers with the current research trends and intuitive data analysis in related research fields in digital form (13). Through literature analysis, it enables the evaluation of scientific output and influence of different countries, institutions, journals, and scholars, providing an invaluable guidance for current research and potential future directions. In the context of cardiovascular diseases, bibliometrics has a significant application.

However, few attempts have been made to assess mitochondrial research in MI using the bibliometric tools such as CiteSpace and VOSviewer. Therefore, in order to understand the beneficial effects and mechanisms of mitochondria in myocardial ischemic diseases and to ascertain current research directions, this study analyzed the relevant literature on MI in the field of mitochondria. First, we identified the cooperation and influence of different authors, countries, institutions, and journals in this field. Then, through the analysis of co-cited references, the basic knowledge and development trend of mitochondrial research in MI were shown. Finally, based on the keyword analysis, the research frontier in the detection of MI was discussed, and insights for the formulation of effective treatment strategies and research plans were provided.

## 2 Methods

### 2.1 Search strategy

The bibliometric analysis was performed using the Web of Science Core Collection (WoSCC) database, including the Science Citation Index Expanded (SCI-Expand 1900-present), the Social Sciences Citation Index (SSCI 1900-present), and the Emerging Sources Citation Index (ESCI 2020-present), selected not only for its extensive repository of leading global academic journals but also for its reported capability to download full citation records. Subsequent to identifying “mitochondria” and “myocardial ischemia” as keywords, in addition to incorporating the MeSH keyword sourced from PubMed, we employed the WoSCC database to extract pertinent studies. This was accomplished through an advanced search strategy, adhering to the search format delineated below: (TS = (“Myocardial Ischemia” OR “Myocard* Ischemia*” OR “Cardiac* Ischemia*” OR “Ischemic Myocardi*” OR “Myocard* Infarction” OR “Myocard* Ischaemia*” OR “Ischemic Heart*” OR “Heart Ischemia*” OR “Ischemic Cardiomyopath*”)) AND (TS = (“Mitochondria*” OR “Mitochondrion*”)). The following were the selection criteria: (1) publication date: January 1, 2015–December 31, 2024, (2) document type: article or review, (3) language: English. All searches were completed and downloaded on the same day (April 17, 2025). The search results were exported in a plain text file format with full record content including cited references selected.

### 2.2 Data collection, analysis, and visualization

**Figure 1:**
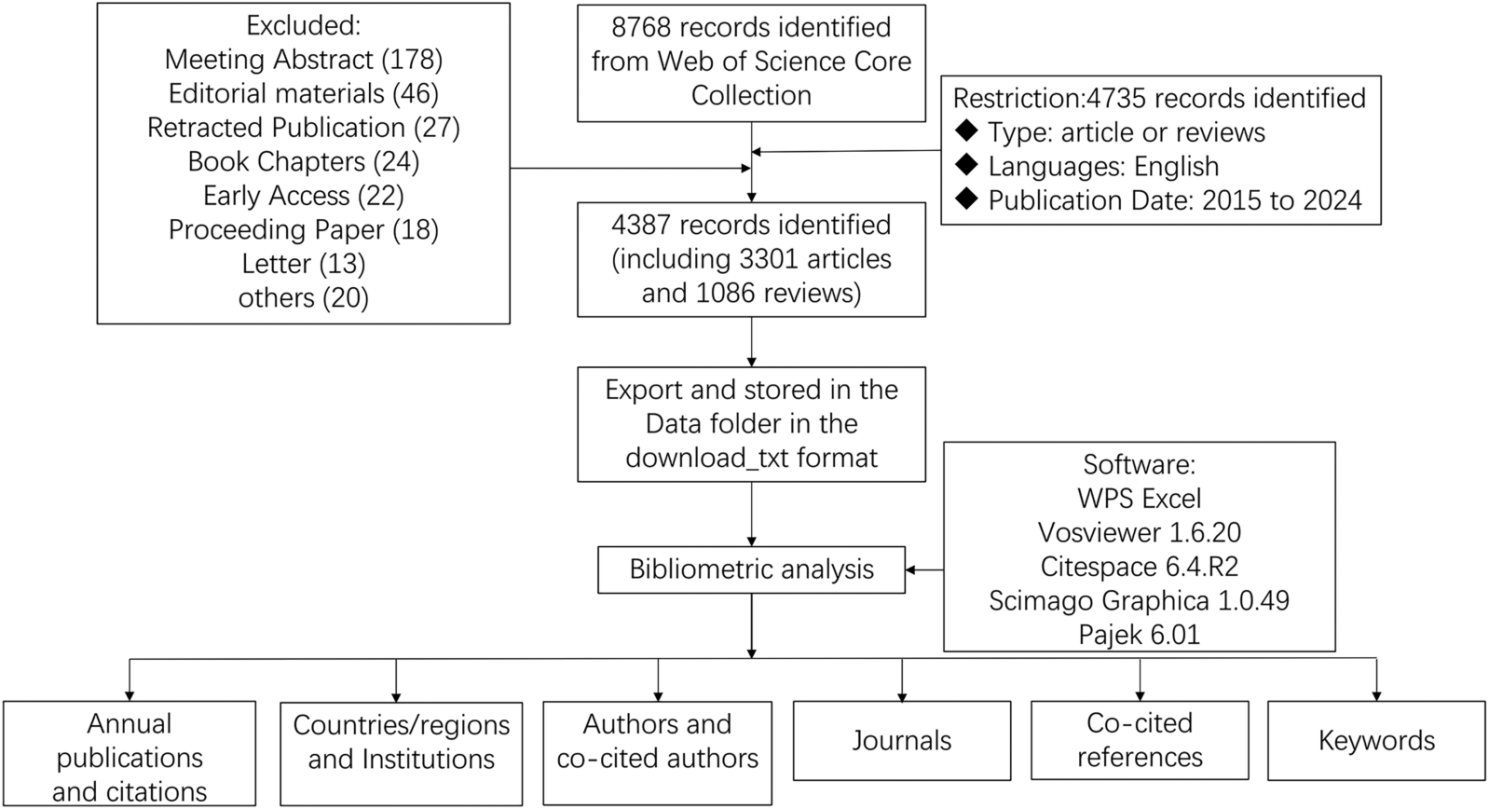
Flowchart for the research's search process.

All data processing was performed manually by the researchers. The search results were independently screened by two investigators, who excluded any irrelevant, duplicate, or withdrawn articles. Any disagreements were resolved through consultation with a third investigator. The WoSCC literature analysis report was employed for data analysis, facilitating the evaluation of publication characteristics such as annual publication output, journal, author, citation frequency, Impact Factor (IF), Journal Citation Reports (JCR), and Hirsch index (H-index). The validated data were subsequently imported into VOSviewer (version 1.6.20), CiteSpace advanced (version 6.4.R2), Scimago Graphica (version 1.0.49), and Pajek (version 6.01) for visual analysis. In essence, this study leverages citation analysis to elucidate the interrelationships among authors, countries, and regions, as well as the associations between institutions and frequently cited references. Furthermore, VOSviewer and CiteSpace were used to display keywords, perform keyword co-occurrence analysis, and perform burst analysis. WPS Excel was used to manage the annual output and to identify research trends in this field. The detailed retrieval processes were illustrated in Figure 1.

## 3 Results

### 3.1 Annual publication growth and citation analysis

**Figure 2:**
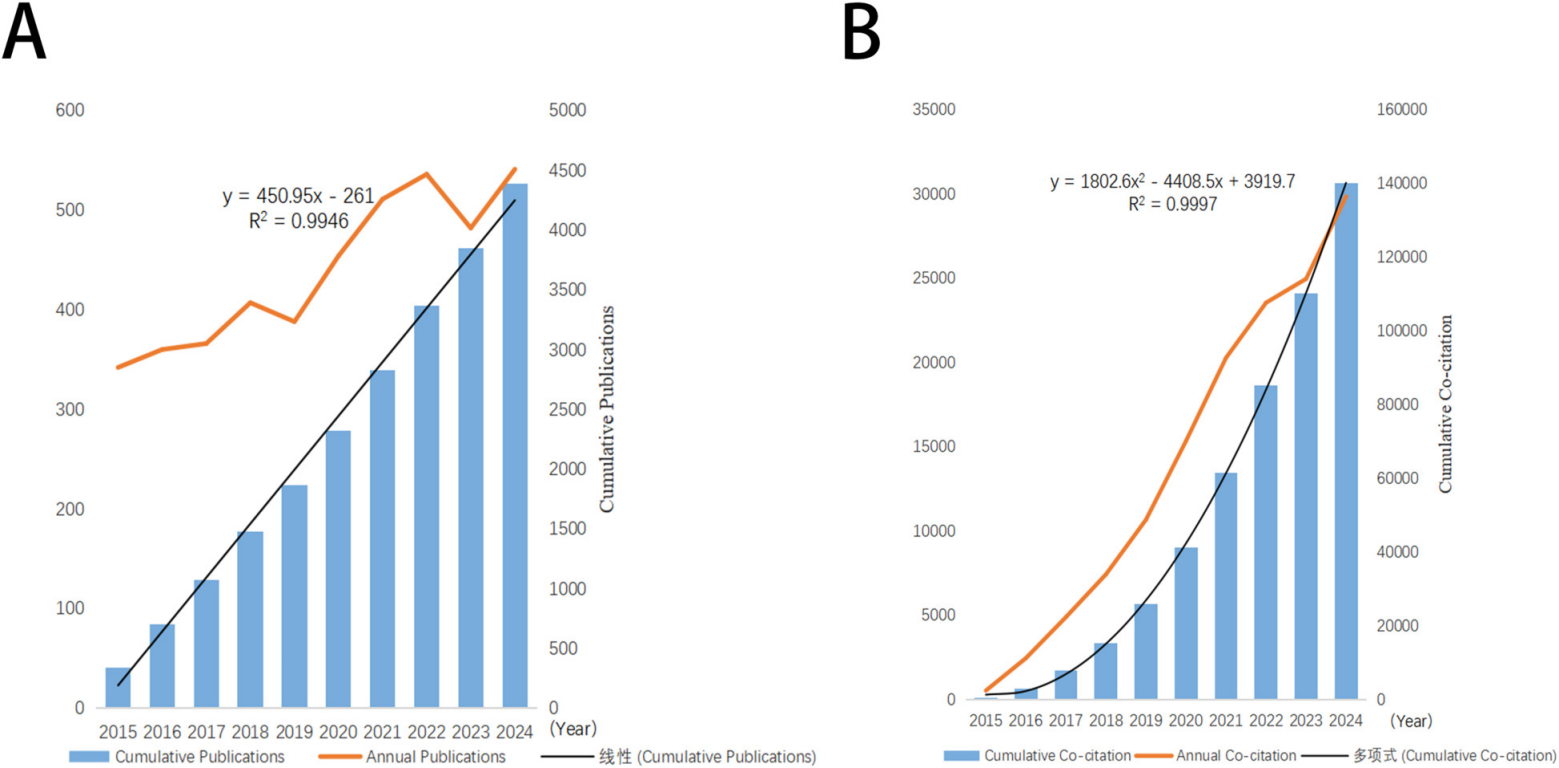
Trends in annual publication and citation output in the field of mitochondria in MI. **(A)** Annual and cumulative number of publications from 2015 to 2024. **(B)** Annual and cumulative number of citations from 2015 to 2024.

In the present study, a comprehensive screening of 4,387 articles was performed about mitochondrial research in MI. The cumulative number of non-self-citations for these articles reached 131,870, equating to an average citation frequency of 33.94 per article. The H-index for the entire article was 146. As depicted in Figure 2A, there was a progressive annual increase in the number of publications from 2015 to 2024. The cumulative co-citation growth curve, as presented in Figure 2B, suggested a logical and promising trajectory for this research field, indicating its potential for sustained development in the future.

### 3.2 Distribution of countries/regions

**Figure 3:**
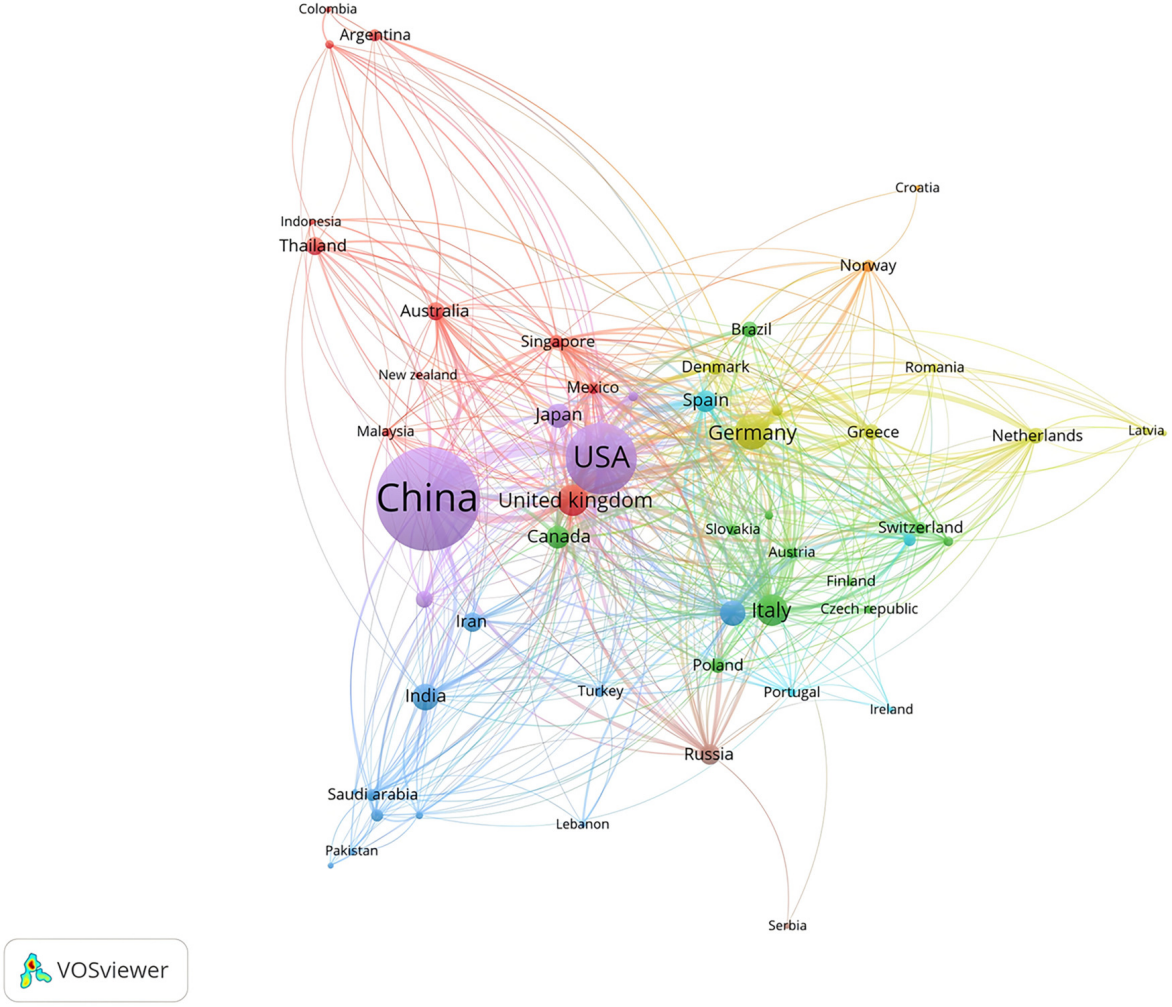
Contribution of different countries/regions to the study of mitochondria in MI from 2015 to 2024.

**Figure 4:**
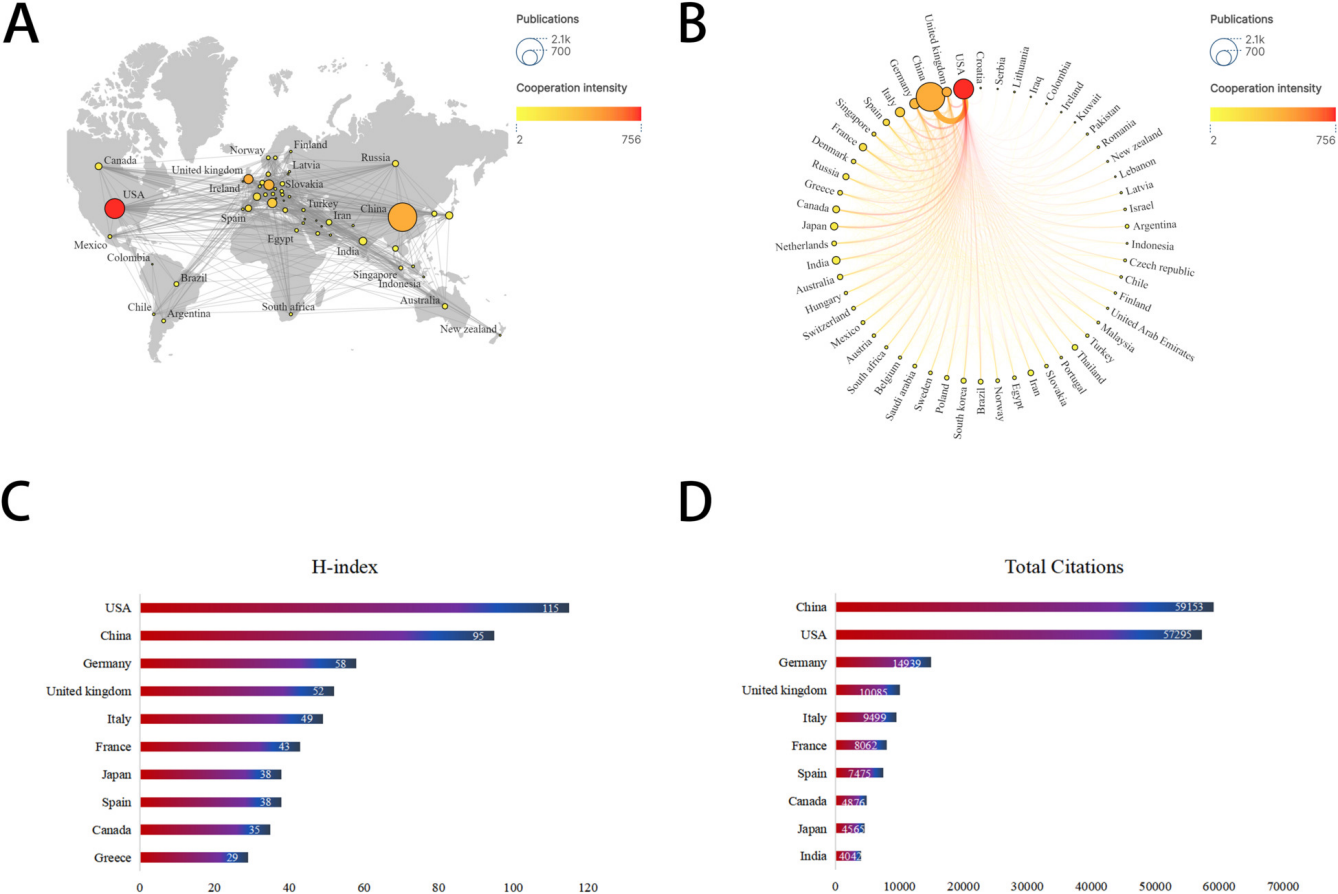
Leading countries/regions related to mitochondria in MI. **(A)** World map of the intensity of collaboration between countries/regions. **(B)** Top 30 countries/regions with most publications. **(C)** Top 10 countries for H-index. **(D)** Top 10 countries for total citations.

As depicted in Figure 3, mitochondrial research articles in the MI have been published by 88 countries or regions over the past decade. The top 10 countries in this field are displayed in Table 1. China led the pack with the highest number of publications (2,093, 47.17%), followed by the United States (1,020, 23.25%) and Germany (263, 5.99%). Considering the total citations, publications from China garnered 59,153 citations, followed by the United States (57,295 citations) and Germany (14,939 citations). Figures 4A,B illustrated that China, the United States, and Europe were the dominant countries and regions for publications. The node color and line thickness indicate the intensity of collaboration between countries, and thicker lines represent closer cooperation, suggesting stronger cooperation between the United States and China. The H-index, a novel method for evaluating academic performance, was also applied to the statistical analysis. The United States led in the H-index, China in the total number of citations, followed by Germany, as shown in Figures 4C,D and Table 1. When these indicators were combined, the United States and China led the field.

**Table 1:** Top 10 countries with the most published studies on mitochondrial research in MI.

Rank	Country	Publications (% of 4,387)	Total citations	Average citation	H-index	Total link strength
1	China	2,093 (47.17)	59,153	28.2623	95	454
2	USA	1,020 (23.25)	57,295	56.1716	115	761
3	Germany	263 (5.99)	14,939	56.8023	58	407
4	Italy	222 (5.06)	9,499	42.7883	49	298
5	United kingdom	221 (5.04)	10,085	45.6335	52	451
6	India	153 (3.49)	4,042	26.4183	28	105
7	France	139 (3.17)	8,062	58	43	198
8	Japan	137 (3.12)	4,565	33.3212	38	132
9	Canada	120 (2.74)	4,876	40.6333	35	135
10	Spain	107 (2.44)	7,475	69.8598	38	206

### 3.3 Authors and co-cited authors

**Figure 5:**
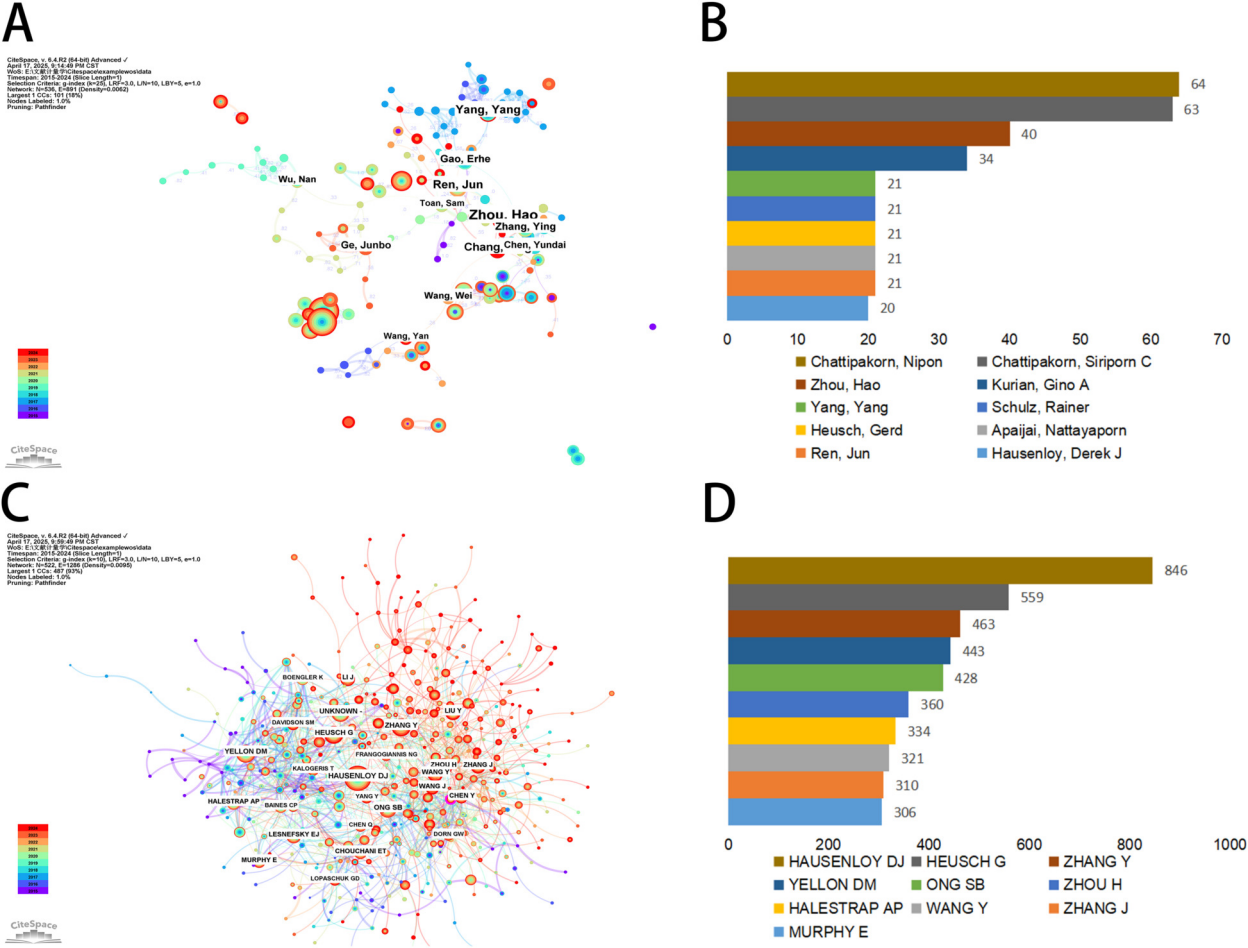
Authors involved in the study of mitochondria in MI. **(A)** Visualization of author co-occurrence based on CiteSpace. **(B)** Top 10 authors in terms by number of publications. **(C)** Visualization of co-cited authors based on CiteSpace. **(D)** Top 10 authors in terms of co-citations.

In the decade following 2015, a sum of 536 authors, each with over two published articles, have conducted research pertinent to mitochondria in MI. The collaborative network of these authors was visualized using CiteSpace, with individual nodes symbolizing an author and the size of the node corresponding to the number of publications. The constructed map (Figure 5A) comprised 536 nodes and 891 edges, exhibiting a network density of 0.0062. The connections between nodes signify collaborations, with the line thickness indicating the degree of collaboration. However, to our surprise, a majority of the authors in this study were found in decentralized nodes, suggesting a need for bolstering collaborations and communications to further drive rapid developments in this field. As depicted in Figure 5B; Table 2, Chattipakorn, Nipon (Chiang Mai University) emerged as the most prolific author with 64 publications, succeeded by Chattipakorn, Siriporn C (63 publications), Zhou, Hao (40 publications), and Kurian, Gino A (34 publications). Notably, the top two collaborative teams hailed from Chiang Mai University. As shown in Figure 5C; Table 2, Hausenloy DJ. topped the co-citations list with 846 citations, followed by Heusch G. (559 citations) and Zhang Y. (463 citations). The leading 10 co-cited authors each had over 306 citations (Figure 5D). Furthermore, centrality is a metric used to assess the importance of an element within the network. When the centrality of an element is greater than 0.1, its relative importance is highlighted with an outer ring (14). We found that the co-cited authors with the highest centrality were Kubli Da. (0.11) and Zhou H. (0.1), indicating that these authors played an important role in bridging gaps in the field.

**Table 2:** Top 10 productive authors and co-cited authors of mitochondrial research in MI.

Rank	Author	Institutions	Publications	Co-cited authors	Institutions	Count
1	Chattipakorn, Nipon	Chiang Mai University	64	Hausenloy, Derek J	National University of Singapore	846
2	Chattipakorn, Siriporn C	Chiang Mai University	63	Heusch, Gerd	University of Duisburg Essen	559
3	Zhou, Hao	Zhejiang University	40	Zhang Ying	Dalian Medical University	463
4	Kurian, Gino A	SASTRA Deemed University	34	Yellon, Derek M	University College London	443
5	Yang, Yang	Air Force Medical University	21	Ong, Sang-Bing	The Chinese University of Hong Kong	428
6	Schulz, Rainer	Justus Liebig University Giessen	21	Zhou, Hao	Chinese Academy of Sciences	360
7	Heusch, Gerd	University of Duisburg Essen	21	Halestrap, Andrew P	University of Bristol	334
8	Apaijai, Nattayaporn	Chiang Mai University	21	Wang Ying	McGill University	321
9	Ren, Jun	Zhongshan Hospital of Fudan University	21	Zhang Jing	University of North Carolina School of Medicine	310
10	Hausenloy, Derek J	National University of Singapore	20	Murphy, Elizabeth	University College London Hospitals NHS Foundation Trust	306

### 3.4 Active institutions

**Figure 6:**
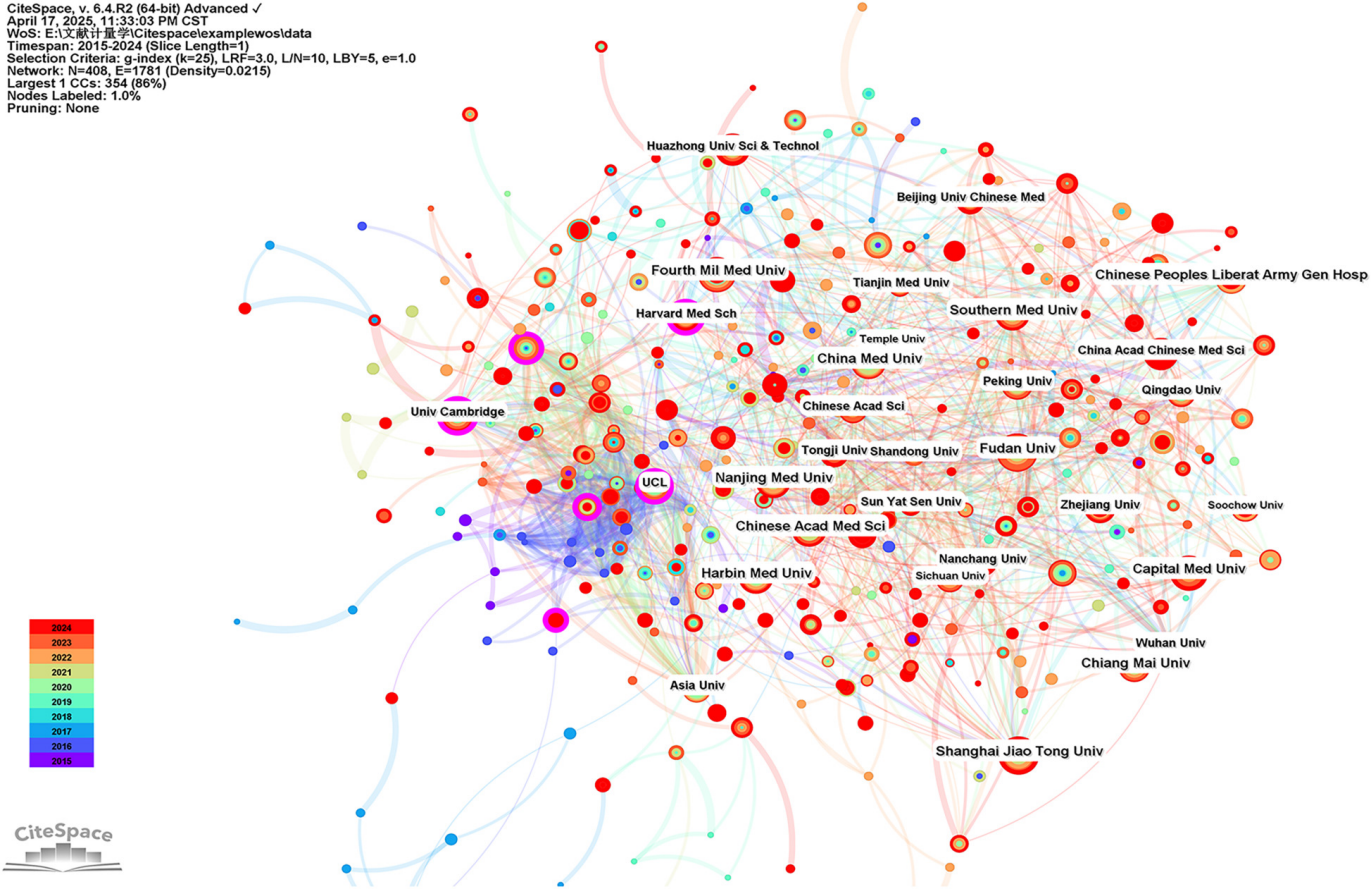
Visualization of institutions carrying out research on the mitochondria of MI.

CiteSpace was used to perform an institutional symbiotic network analysis to find organizations or institutions with relatively mature research. Nodes in the graph represent institutions, with larger nodes representing more publications from the institution; links between nodes indicate collaborations between institutions, the color of the link indicates the start of the collaboration, and the thickness of the line indicates the strength of the collaboration. Table 3 listed the top 10 institutions in terms of number of publications and centrality of mitochondria in MI research. In this field, the Fourth Military Medical University (86 publications) led in the number of publications, followed by Fudan University (83 publications) and Shanghai Jiao Tong University (71 publications). As shown in Figure 6, more circles outside the nodes indicate a higher degree of centrality, with centrality ≥0.10 indicating that these institutions act as bridges and may lead to transformative discoveries. As shown in Table 3, the top three institutions in terms of centrality were Inserm (0.22), the University of Cambridge (0.19), and Harvard University (0.16). The results suggest that these three institutions worked closely with others and played an important role as intermediary platforms in the development of the mitochondrial field of MI.

**Table 3:** Top 10 institutions of mitochondrial research in MI by count and centrality.

Rank	Institutions	Count	Rank	Institutions	Centrality
1	Air Force Medical University	86	1	Inserm	0.22
2	Fudan University	83	2	University of Cambridge	0.19
3	Shanghai Jiao Tong University	71	3	Harvard University	0.16
4	China Medical University	70	4	University College London	0.13
5	Chiang Mai University	68	5	Harvard Medical School	0.12
6	Harbin Medical University	67	6	Semmelweis University	0.11
7	Capital Medical University	62	7	Chinese Academy of Sciences	0.1
8	Chinese Academy of Medical Sciences	61	8	National University of Singapore	0.1
9	Southern Medical University	59	9	The University of Hong Kong	0.1
10	Nanjing Medical University	58	10	Asia University	0.08

### 3.5 Core journals

**Figure 7:**
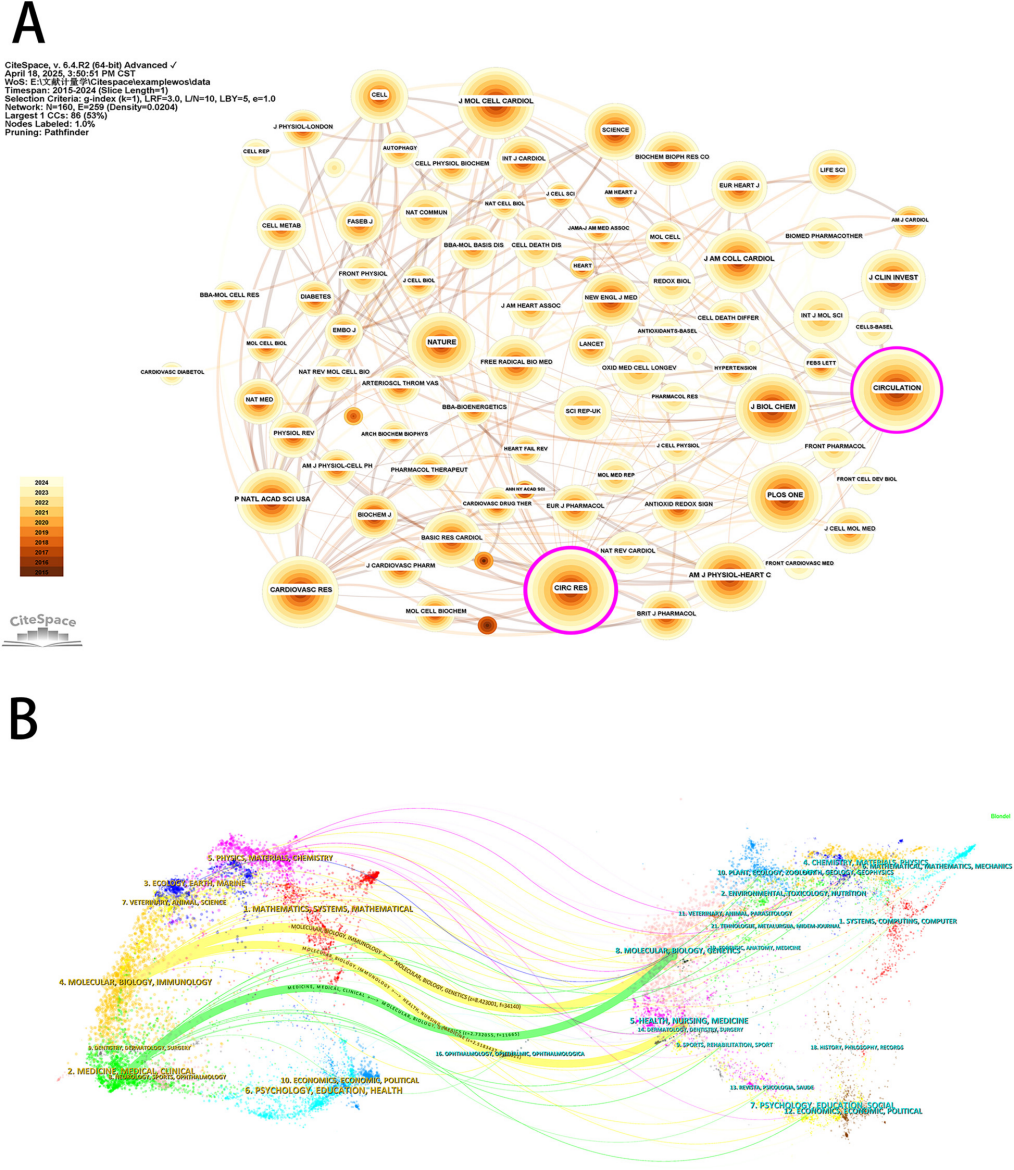
Journal analysis for mitochondrial research in MI. **(A)** Visualization of journals based on CiteSpace. **(B)** The dual-map overlay of journals based on CiteSpace.

Analysis of the sources of the included literature showed that the *International Journal of Molecular Sciences* (143 publications) was the journal with the highest number of publications in this field, followed by *Oxidative Medicine and Cellular Longevity* (94 publications) and *Biomedicine & Pharmacotherapy* (84 publications) (Table 4). All of the top 10 journals in terms of publication volume were in the first or second (Q1 or Q2) JCR partition in 2024, suggesting a high degree of reliability in the quality of the publications included in this study. This information may guide researchers to prioritize these journals for their submissions. The co-citation analysis of the journals showed that *Circulation Research* (3,066 total citations) was the most cited journal, followed by *Circulation* (3,065 total citations) and *Cardiovascular Research* (2,568 total citations) (Figure 7A). In 2024, *Circulation Research* had an IF score of 16.2, the highest among the top 10 most cited journals. And more than half of the top 10 most cited journals were categorized as Q1. Furthermore, the journal with the highest centrality was *Circulation Research* (0.26) (Table 4), which also indicates that these journals have a higher impact in the field. The dual maps of CiteSpace reflect the development of research in different disciplines. Citing articles are shown on the left, cited articles are shown on the right, and the colored curved paths in the middle indicate citation relationships. As shown in Figure 7B, the orange citation paths indicated that research in molecular/biology/genetics journals and health/nursing/medicine journals was frequently cited by molecular/biology/immunology journals. The green citation path indicated that research in molecular/biology/genetics journals was frequently cited by medicine/medical/clinical journals. Meanwhile, the edge region of the overlay graph showed that the citation paths of systems/computing/computer, environmental/toxicology/nutrition, earth/geology/geophysics, and other disciplines were also involved in the study of mitochondria and MI. This suggests that researchers have conducted multidisciplinary and collaborative studies in this field.

**Table 4:** Top 10 most commonly cited journals in the field of mitochondrial research in MI.

Rank	Journal	Count	IF_2024_	JCR_2024_	Rank	Journal	Centrality	IF_2024_	JCR_2024_
1	*International Journal of Molecular Sciences*	143	4.9	Q1/Q2	1	*Circulation Research*	0.26	16.2	Q1
2	*Oxidative Medicine and Cellular Longevity*	94	/	/	2	*Circulation*	0.1	38.6	Q1
3	*Biomedicine & Pharmacotherapy*	84	7.5	Q1	3	*Cardiovascular Research*	0.06	13.3	Q1
4	*Basic Research in Cardiology*	78	8	Q1	4	*Journal of Biological Chemistry*	0.04	3.9	Q2
5	*Journal of Molecular and Cellular Cardiology*	73	4.7	Q1/Q2	5	*Proceedings of The National Academy of Sciences of The United States of America*	0.04	9.1	Q1
6	*Frontiers in Pharmacology*	68	4.8	Q1	6	*International Journal of Molecular Sciences*	0.04	4.9	Q1/Q2
7	*Frontiers in Cardiovascular Medicine*	66	2.9	Q2	7	*Oxidative Medicine and Cellular Longevity*	0.04	/	/
8	*European Journal of Pharmacology*	65	4.7	Q1	8	*American Journal of Physiology-Heart and Circulatory Physiology*	0.03	4.1	Q1
9	*Scientific Reports*	61	3.9	Q1	9	*Diabetes*	0.03	7.5	Q1
10	*Free Radical Biology and Medicine*	57	8.2	Q1	10	*Biomedicine & Pharmacotherapy*	0.03	7.5	Q1

### 3.6 Co-cited references

**Figure 8:**
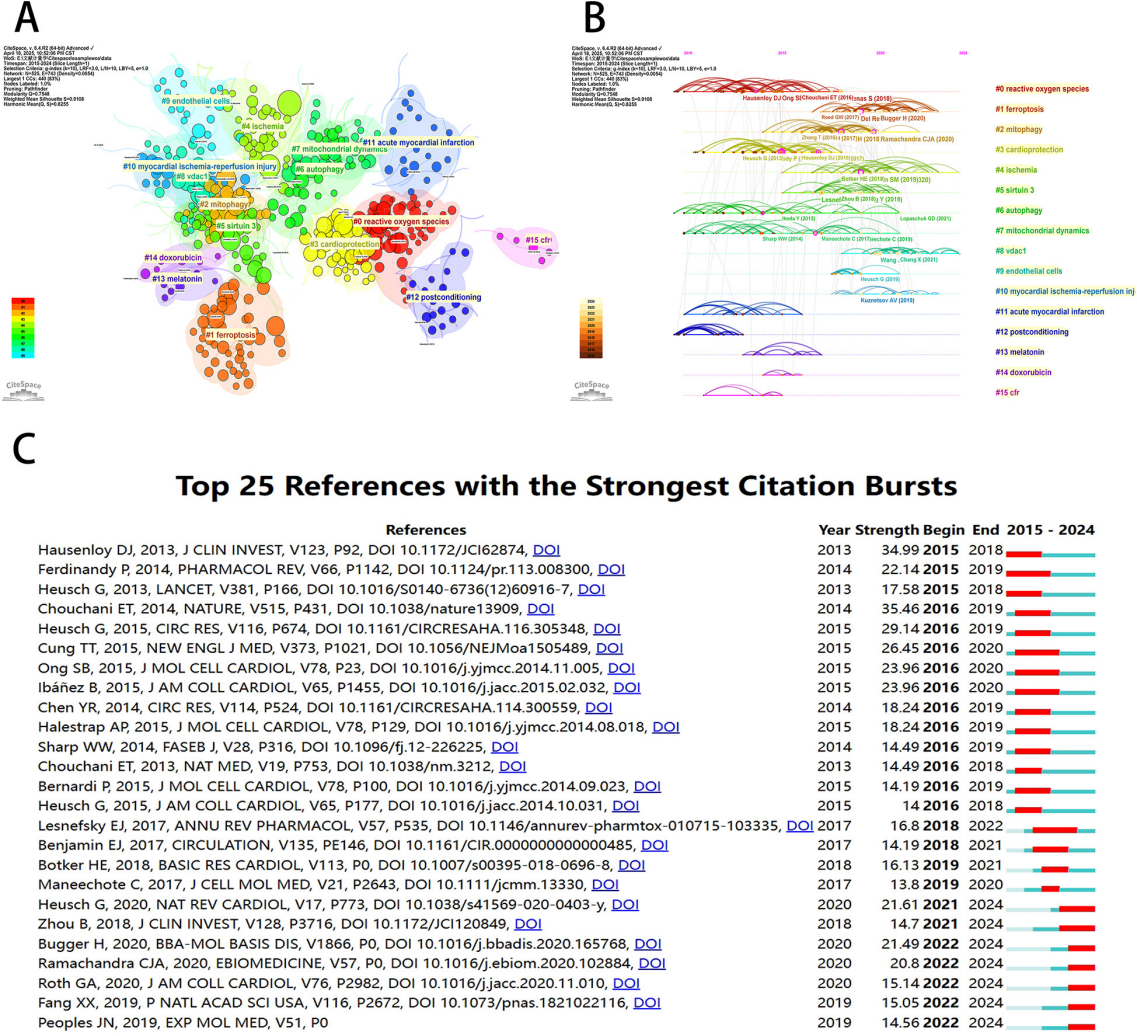
Visualization of co-cited references for mitochondrial research in MI by the CiteSpace software. **(A)** Visualization of cluster analysis of co-cited references. **(B)** Timeline graph of the 16 cluster analysis. **(C)** The top 25 references with the strongest citation bursts.

Co-cited reference detection can be used to identify trends and changes in citation patterns over time by detecting a decrease or increase in specific co-cited studies. Co-cited references indicate the degree of relationship between references, revealing key literature, knowledge structures, and promising directions in the field. Since the network was dense and the centrality cannot be calculated, we set the parameters as follows: time slice (2015–2024), selection criteria (k = 10), and pruning in the CiteSpace software. As shown in Figure 8A, a symbiotic network with node number 525, connection number 743, and density 0.0054 was obtained. We then performed a cluster analysis of the cited references based on the log-likelihood (LLR) algorithm, and 16 clusters were shown in Figure 8B. The value of the cluster number indicates the intensity of focus on the clustered topic within the discipline. A lower cluster value corresponds to a higher level of attention. Among them, the cluster modularity Q = 0.7548 and the average silhouette score S = 0.9108 indicated that the clustering results were reasonable and the cluster structure was significant. According to Figure 8B, cluster #0 (reactive oxygen species), cluster #3 (cardioprotection), cluster #6 (autophagy), cluster #7 (mitochondrial dynamics), cluster #11 (acute myocardial infarction), and cluster #12 (postconditioning) were initiated earlier. On the other hand, cluster #4 (ischemia) and cluster #8 (vdac1) are still at the forefront of research.

As shown in Figure 8C, CiteSpace focused on the 25 most cited references in the field of mitochondrial research on MI in the last decade. Among them, the earliest cited reference was a 2013 *Journal of Clinical Investigation* (IF 2024 = 13.6) article by Hausenloy DJ et al. titled “Myocardial ischemia-reperfusion injury: a neglected therapeutic target” (15). The paper provided a comprehensive overview of the mechanical and pharmacological adjunctive measures to mitigate myocardial reperfusion injury, underscoring the centrality of myocardial reperfusion in the treatment of acute myocardial infarction injury.

### 3.7 Keyword analysis

**Figure 9:**
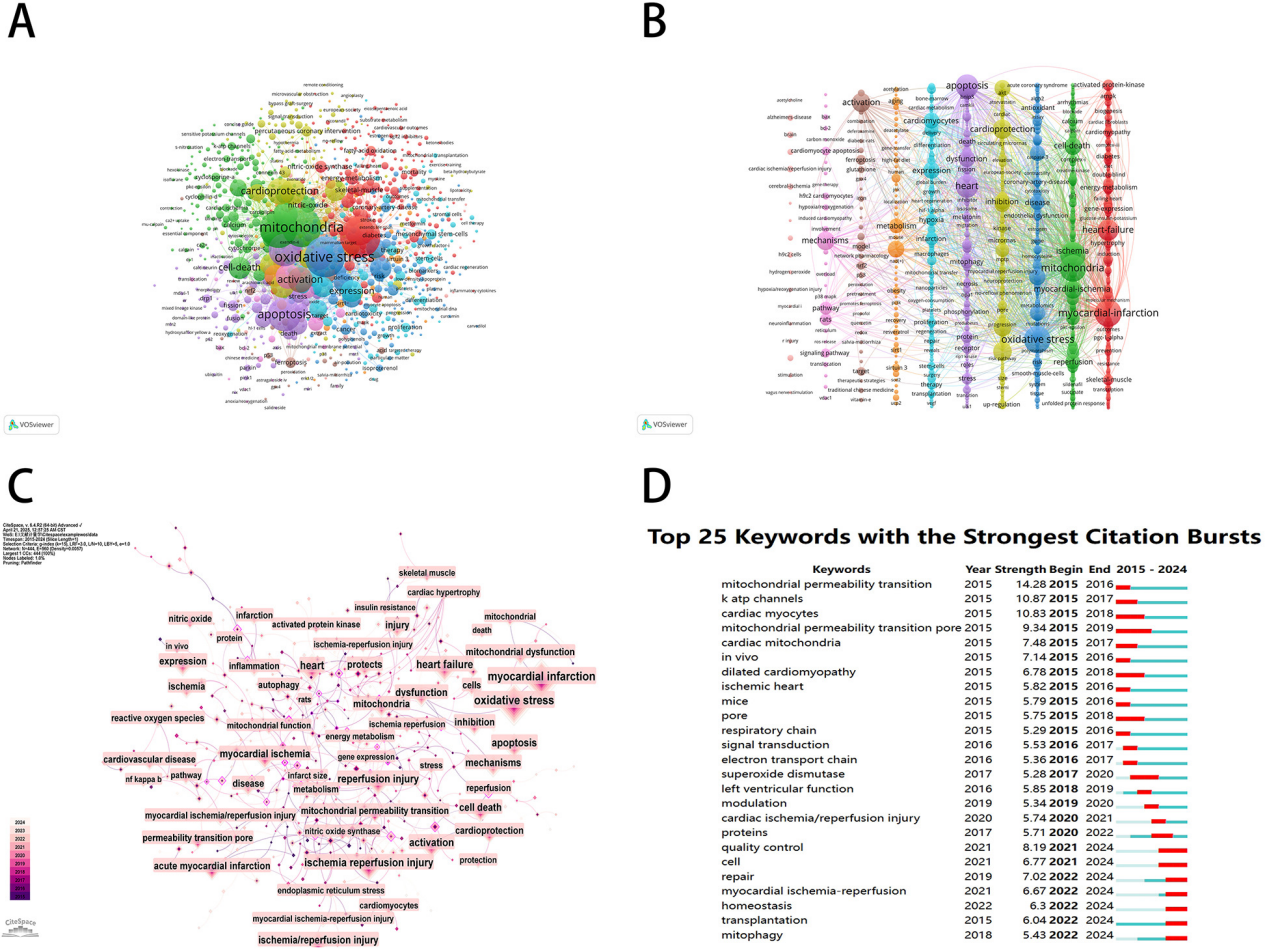
Keyword mapping of mitochondrial research in MI. **(A)** Keyword co-citation network based on VOSviewer. **(B)** Visualization of keyword clustering in the vertical form (divided into 10 clusters according to different colors). **(C)** Visualization of keywords based on CiteSpace. **(D)** The top 25 keywords with the strongest citation bursts.

A keyword co-occurrence network provides a comprehensive view of current research hotspots and trends in a specific field. We employed VOSviewer software to perform a keyword analysis, and after merging the similar keywords, we extracted 1,330 keywords with a minimum of five occurrences (Figure 9A). As shown in Figure 9B, a subsequent cluster analysis of keywords yielded ten clusters. The largest cluster (red) contained 199 keywords, including myocardial infarction, heart failure, mitochondrial-function, activated protein-kinase, energy-metabolism, skeletal-muscle, nitric-oxide synthase, endoplasmic-reticulum stress, gene-expression, insulin-resistance, and cardiomyopathy. The second category (green) contained 170 keywords, including mitochondria, ischemia-reperfusion injury, myocardial ischemia, cell-death, permeability transition pore, reactive oxygen species, nitric-oxide, rat-heart, and calcium. The third category (blue) contained 144 keywords, including oxidative stress, inflammation, cardiovascular-disease, mitochondrial dysfunction, disease, nf-kappa-b, antioxidant, atherosclerosis, and *in vivo*. The fourth category (yellow) contained 134 keywords, including cardioprotection, reperfusion injury, protects, acute myocardial-infarction, and inhibition. The fifth category (purple) contained 116 keywords, including apoptosis, heart, dysfunction, autophagy, mitophagy, protein, stress, and receptor. The sixth category (aqua green) contained 104 keywords, including expression, cardiomyocytes, cells, infarction, hypoxia, therapy, mesenchymal stem-cells, and angiogenesis. The seventh category (orange) contained 54 keywords, including myocardial ischemia/reperfusion injury, metabolism, aging, sirtuin 3, obesity, and sirt1. The eighth category (brown) contained 33 keywords, including activation, model, ferroptosis, target, nrf2, lipid-peroxidation, acid, glutathione, p53, and iron. The ninth category (peach) contained 28 keywords, including mechanisms, pathway, rats, signaling pathway, cardiomyocyte apoptosis, h9c2 cardiomyocytes, involvement, and hypoxia/reoxygenation. The tenth category (pink) mainly included brain, cardiac ischemia/reperfusion injury, and rat model. In addition, we used the CiteSpace software to visually represent the keywords, which generated a map with 444 nodes, 560 connections, and a density of 0.0057. With the exception of “mitochondria” and “myocardial ischemia,” oxidative stress emerged as the predominant term (*n* = 1221), closely followed by apoptosis (*n* = 828) and cardioprotection (*n* = 530) in Figure 9C. According to Figure 9D, mitochondrial permeability transition showed the most intense bursts (strength = 14.28), followed by k-ATP channels (strength = 10.87) and cardiac myocytes (strength = 10.83). It is noteworthy that in the study topics “mitophagy” (2022–2024), “transplantation” (2022–2024), and “homeostasis” (2022–2024) exhibited citation bursts until 2024, indicating that these areas are novel research topics recently.

## 4 Discussion

In recent decades, the morbidity and mortality of myocardial ischemia-related diseases have remained high and have become an important issue for global medicine (16, 17). Mitochondria, as a key regulatory target of various physiological and pathological processes (12, 18–22), play indispensable roles in normal myocardial function and disease processes, making mitochondrial research in MI a rapidly expanding field. Our analysis has revealed that over the past decade, researchers worldwide have focused on mitochondrial mechanisms to alleviate myocardial ischemic diseases. These efforts encompass ischemic heart disease, dilated cardiomyopathy, and myocardial I/R injury. In summary, these studies of articles aimed to restore mitochondrial functions in MI by targeting pathophysiological mechanisms such as metabolic disorders, oxidative stress, cell death, hypoxia, inflammation, endothelial cell dysfunction, and drug-induced cellular damage; core mitochondrial processes including permeability transition, electron transport chain, mitophagy, and mitochondrial dynamics. These studies were based on different molecular mechanisms, such as the sirtuin-3 signaling pathway, VDAC1 pathway, caspase-3 pathway, and AMPK/PGC-1α pathway. These therapeutic targets reflect the multiple effects of mitochondria in MI. Consequently, researchers employed animal models to validate the role of maintaining mitochondrial homeostasis in ameliorating MI. These research findings also included several promising therapeutic strategies, including traditional Chinese medicine (TCM), stem cell therapy, and multi-omics analysis. Chen et al. utilized Yiqi Huoxue prescription to alleviate MI and ischemia-reperfusion (I/R) injury by targeting mitophagy (23). Ikeda G et al. improved cardiac function by restoring myocardial bioenergetics by harvesting and injecting mitochondria-rich EVs into the myocardium (24). Ranjbarvaziri S et al. performed a comprehensive multiomics profile of the molecular (transcripts, metabolites, and complex lipids), ultrastructural, and functional components of hypertrophic cardiomyopathy energetics using myocardial samples from clinical patients, and the results suggested that metabolic signaling disorders and mitochondrial dysfunction are common pathogenic mechanisms in patients with hypertrophic cardiomyopathy (25). Overall, articles in this field continued to focus on the innovativeness of mitochondrial mechanisms and the relevance of pharmacological effects. On the other hand, reviews in this field have focused primarily on the pathophysiological mechanisms and signaling pathways related to mitochondrial research in MI. These articles have described the key targets of mitochondria in myocardial ischemic injury and protection from different perspectives. The dynamic changes, metabolic regulation, and death signal integration roles of these targets determine the fate of cardiomyocytes. In conclusion, these studies have provided an overview and direction for research on the link between mitochondria and MI, and intervention strategies targeting these processes have been an important direction for cardioprotection.

### 4.1 General information about MI in the mitochondrial field

This study analyzed 4,387 retrieved articles based on the WoSCC database using CiteSpace, VOSviewer, Excel, Scimago Graphica, and Pajek with a focus on mitochondrial research in the field of MI. The aim was to identify key research directions and emerging trends in the field. The comprehensive analysis revealed a steady annual growth in publications over the last decade, with a significant acceleration since 2015. This trajectory suggests that mitochondrial research in MI has been innovative and relatively mature over the last decade. Furthermore, our analysis revealed a total of 4,387 publications from 2015 to 2024, consisting of 3,301 articles (75.25%) and 1,086 reviews (24.75%), distributed across 202 academic journals, affiliated with 408 institutions, and spanning 88 countries or regions. Given the current number of publications worldwide, a robust developmental momentum for mitochondria in the field of MI is expected in the coming years (26, 27).

Using CiteSpace and VOSviewer software, we performed a bibliometric analysis to analyze mitochondrial research in MI. Further analysis identified the United States, China, and major European countries as the major contributors to the field. China contributed the highest number of publications and citation frequency, while the United States led in H-index compared to other countries or regions. In particular, the United States was dominant in terms of authorship and international collaboration. A close review of the research conducted in the United States revealed an emphasis on the integration of basic research with clinical translation (28, 29), which may be an important reason why the United States has been a leader in the field of mitochondrial therapeutic targets and MI. In the United States, researchers have focused on the innovative use of novel testing techniques and tools. Lin et al. developed an artificial mitochondrial transplantation strategy that transiently enhanced EC bioenergetics and enabled them to form functional vessels in ischemic tissue without the support of mesenchymal stromal cells (30). Similar research can effectively combine molecular biology, materials science, and engineering with cardiovascular disease research and foster interdisciplinary and multiregional collaboration. Furthermore, the United States was home to a number of internationally recognized pharmaceutical companies with an advantage in drug development and preclinical research. On the other hand, the European countries have shown a significant interest in investigating the role of mitochondria in the physiopathological mechanisms of MI. Researchers in this field have demonstrated a high level of competence in conducting and integrating large-scale sample analyses (31, 32). These analyses have facilitated the identification and application of more valuable and representative biomarker indicators (33, 34). Research in China has traditionally focused on the complex relationship between mitochondria and MI. However, despite the substantial number of publications in this field, diagnostic and testing methods have often been limited by standards and guidelines established by Western countries. Currently, China is actively exploring additional therapeutic strategies based on mitochondrial targeting, such as exploring the potential and unique advantages of TCM to modulate mitochondrial homeostasis to improve MI (35). At the same time, Chinese researchers are actively promoting the translation of basic cardiovascular research into clinical applications (36, 37). In addition, initiatives have been proposed and implemented to actively expand international and regional research collaborations, establish partnerships with pharmaceutical companies, strengthen medical industrialization research and development to meet the dual challenges of project demand and funding, and introduce innovative scientific tools and methods to promote innovation in clinical diagnosis and treatment and basic research.

A review of IF and journal centrality indicated that *Circulation Research* was a high-quality publication with significant impact in the field between MI and mitochondria. It should be noted that journals with high publication counts may not be significantly influenced by related research areas. In this study, the top three most influential co-cited journals with IFs greater than 10 were C*irculation Research*, *Cell Death and Differentiation*, and *Redox Biology*. As a result, when tracking the research hotspots and significant achievements in the field, these can be found in high-quality journals. The analysis also revealed that the top three authors were from Asian countries, with nine of the top ten institutions being Chinese, underscoring China's significant contribution to mitochondrial research in MI over the past decade. Co-citation pattern analysis revealed that David J. Hausenloy (National University of Singapore), Gerhard Heusch (University of Duisburg-Essen), and Zhang Ying (Dalian Medical University) emerged as the three most frequently co-cited authors, underscoring their substantial contributions and esteemed academic reputations in the field.

Highly cited references serve as cornerstones in certain fields of research. By analyzing the co-cited references, it is possible to quickly grasp the knowledge background of the field and to identify the focus and research trends of mitochondrial research over the past decade. In addition, the combination of co-cited references with keyword clustering helps to explore the real hotspots in the field based on current topics. When evaluated in conjunction with the most recent highly cited references, three references have received significant attention. The most influential publication by Heusch G, entitled “Myocardial ischaemia-reperfusion injury and cardioprotection in perspective,” was published in *Nature Reviews Cardiology* in 2020 (38). This review discussed the translational gap between experimental I/R models and clinical outcomes, and highlighted the pathophysiological significance of autophagy regulation and dependent cell death modalities. It also advocated therapeutic strategies targeting non-cardiomyocyte populations and long-term outcomes, including infarct resolution and reverse remodeling. In 2020, Ramachandra CJA and his colleague published a review entitled “Mitochondria in acute myocardial infarction and cardioprotection” in *Ebiomedicine* (12). This review systematically analyzed the dual role of mitochondria as injury amplifiers and therapeutic targets. It showed how mitochondrial dysfunction manifests as energy depletion, calcium overload, overproduction of reactive oxygen species, and activation of the mitochondrial permeability transition pore (MPTP), which determines the fate of cardiomyocytes. These findings were further confirmed by Bugger H's 2020 study “Mitochondrial ROS in myocardial ischemia reperfusion and remodeling” (39), which additionally elucidated the pattern of reactive oxygen species (ROS) generation from the acute I/R injury to chronic post-infarction remodeling. This work demonstrated that mitochondrial-derived oxidants not only damage electron transport chain components and mtDNA, but also activate inflammatory cascades, extracellular matrix modification pathways, and pro-apoptotic signals that collectively drive maladaptive ventricular remodeling. In conclusion, these studies represent a significant advance in the field by transcending the limitations of previous pathological understanding in this field and comprehensively analyzing the complexity of the mitochondrial network in MI. In addition, these studies provide a critical assessment of the discrepancy between preclinical and clinical findings and the current understanding of how to diagnose and treat MI.

### 4.2 Research hotspots and emerging frontiers

The use of “top keywords with the strongest citation bursts” can indicate the popularity and relevance of specific research topics. In the field of mitochondria in MI, the CiteSpace software analyzed the major research areas, including “mitophagy,” “homeostasis,” “ischemia,” and “transplantation.” The analysis revealed a shift in research focus from mitochondrial function alone to the multiple causal pathways of mitochondrial injury. The scope of research has expanded to include the study of intercellular communication involving endothelial cells, immune cells, and smooth muscle cells (40). In addition, research has expanded to include the study of ferroptosis and apoptosis in the context of cell death (41, 42). In terms of research methodology, multi-omics studies and multidisciplinary collaborations are also gradually increasing (43), which will further facilitate the clinical translation and application of research in this field.

### 4.3 The role of mitochondrial research in MI

Mitochondria, as the primary site of aerobic respiration in eukaryotes, play both a role in cardioprotective effects and a major source of myocardial injury (12). Mitochondrial quality control, including mitochondrial oxidative stress, mitochondrial autophagy, mitochondrial dynamics, and mitochondrial biogenesis, is a central aspect of maintaining mitochondrial homeostasis (44, 45). Molecular oxygen, as the terminal receptor of the electron transport chain, ensures the homeostasis of the energy metabolism network by supporting the formation of the mitochondrial membrane potential, which in turn regulates the cell cycle and biogenesis (46). Hypoxia, a pathological state characterized by reduced oxygen tension, triggers the production of large amounts of free radicals and ROS by the ETC complex (47). ROS in this state damage non-specifically various cellular macromolecules, cell membranes, and mitochondria themselves, resulting in inhibition of mitochondrial biogenesis, alteration of mitochondrial permeability and membrane potential, accompanied by the abnormalities of mitophagy and mitochondrial dynamics under stress conditions (48, 49). Mitochondrial morphological and functional changes accompany the entire process of cardiac physiopathological development, so it is valuable to explore the role of mitochondria at each stage of ischemic preconditioning, postconditioning, and distal ischemic adaptation in order to improve the prognosis of MI (50–52). Furthermore, it is imperative to investigate the morphology and function of mitochondria due to the alterations in mitochondrial structure and function induced by myocardial ischemic injury, which directly result in impaired energy metabolism in cardiomyocytes (53). A recent study on mitochondria in 2024 found that mitochondria may have a mitochondrial morphological change that is not dependent on mitochondrial fusion and fission (54, 55), a breakthrough that has changed the understanding of fission- and fusion-dependent mitochondrial dynamics from previous studies. That is why scientists around the world continue to explore and discover the frontiers of this field.

### 4.4 The role of TCM in mitochondrial MI research

Currently, the main treatment for MI is medication or percutaneous coronary intervention to stop blood clots, but this does not effectively relieve symptoms and side effects such as chest pain, shortness of breath, or I/R injury. TCM is an advantageous discipline in the treatment of MI, reducing clinical symptoms and cardiovascular adverse events (56). The basic pathogenesis of myocardial ischemia is deficiency, and the basic symptoms are qi deficiency, blood stasis, and loss of support to the heart and veins. Mitochondria, as the power source and energy supplier of cells, are highly compatible with “qi” in TCM. The operation of qi drives the process of energy metabolism and generates the power to maintain the functional activities of the organism (57). When the heart qi is weak or insufficient, it is unable to propel the blood to flow, resulting in blood vessel stagnation and poor heart structure and function, which further aggravates myocardial injury (58). Many studies have shown that TCM, such as herbal compound preparations, crude extracts, and active monomers, is widely used to induce mitophagy through various pharmacological mechanisms and signaling pathways as a therapeutic approach for cardiovascular diseases. They represent a hotspot for basic cardiovascular research and a promising source for future drugs (59, 60). Dong et al. elucidated the effects of the Yixin formula in warming yang energy, activating blood circulation, and eliminating blood stasis, and outlined the cardioprotective effects of this formula in reducing oxidative stress damage and apoptosis in ischemic cardiomyopathy rats (61). Hu et al. demonstrated that a ginseng tRNA fragment protected the heart from I/R injury by targeting the lncRNA MIAT/VEGFA pathway, thereby maintaining cytoskeletal integrity and mitochondrial function in cardiomyocytes (62).

The strengths of TCM in this field come from its multi-targeted effects, including anti-inflammatory, antioxidant, and cardioprotective properties, as well as its natural composition and minimal side effects. However, its theoretical structure differs from that of modern medicine, which hinders understanding and recognition by mainstream scholars. Furthermore, the implementation of personalized TCM treatment may pose a significant challenge to the experimental argumentation framework. Therefore, further research on TCM in this field is necessary and highly anticipated.

## 5 Limitations

This study is the first to present a bibliometric study of mitochondria in MI. Despite these findings, the study is not without limitations. The cut-off date for the current study survey was set at December 31, 2024, but new studies were excluded from the final bibliometric collection due to continued publication in the WOS database. Due to limitations in the software and methodology used, the analysis was restricted to articles and reviews, which may have resulted in a certain degree of neglect. In addition, differences in the quality of publications in the databases may have reduced the precision of the bibliometric analyses.

## 6 Conclusions

In this study, bibliometric analysis was used to provide a comprehensive overview of mitochondrial research in MI. Over the past decade since 2015, the number of Science Citation Index papers focusing on MI in mitochondrial research has increased year on year, with China and the United States remaining the leaders in the field. Hausenloy, Derek J., of the National University of Singapore, is one of the most influential scientists in the field. Despite the abundance of research on MI and mitochondria, translation into clinical drug development has been slow. We advocate the use of a variety of models and methods for more systematic, rigorous, and scientific research and evaluation that can be used for basic research and drug development of MI in the mitochondrial field.

## Data Availability

The original contributions presented in the study are included in the article/Supplementary Material, further inquiries can be directed to the corresponding author.
