# Supplementary material for: A bibliometric and visualized analysis of research on mitochondria in myocardial ischemia from 2015 to 2024
Source: Front Cardiovasc Med. 2025 Jul 7;12:1547604. doi: 10.3389/fcvm.2025.1547604 (PMC12277348; doi:10.3389/fcvm.2025.1547604)
Supplement: Supplementary file 1 [file Table1.doc]

Supplementary Material

# Supplementary Information: Search Terms Implementation

**Supplementary Table 1**

| **Rank** | **Search terms** | **Source basis** |
| --- | --- | --- |
| #1 Collection of search terms | "Myocardial ischemia", "Mitochondria", "Cardiac", "Myocardi", "Myocard", "Heart", "Ischemic", "Ischemias", "Ischemia", "Ischaemia", "Infarction". "Cardiomyopath", "Mitochondrial", "Mitochondrion" | Bibliometric articles |
| #2 Standardization of search terms | "Myocardial Ischemia", "Ischemia", "Mitochondria", "Heart" | Mesh Thesaurus (https://www.ncbi.nlm.nih.gov/mesh/?term=) |
|  | "Cardiac", "Myocard", "Ischemic", "Myocardi", "Cardiomyopath", "Infarction", "Ischaemia" | CNKI Translation Assistant (http://dict.cnki.net/) |
| #3 Merge synonyms | "Ischemias" is merged and unified as "Ischemia" or "Ischaemia" | Mesh Thesaurus (https://www.ncbi.nlm.nih.gov/mesh/?term=) |
|  | "Mitochondrial" is merged and unified as "Mitochondria" or "Mitochondrion" | Mesh Thesaurus (https://www.ncbi.nlm.nih.gov/mesh/?term=) |
| #4 Identify the search terms | "Myocardial Ischemia", "Myocard* Ischemia*", "Cardiac* Ischemia*", "Ischemic Myocardi*", "Myocard* Infarction", "Myocard* Ischaemia*", "Ischemic Heart*", "Heart Ischemia*", "Ischemic Cardiomyopath*" (* is a wildcard) | #1, #2, #3 |
|  | "Mitochondria*", "Mitochondrion*" (* is a wildcard) |  |

# Supplementary Information: Search Strategy Implementation

**2.1 Web of science：**SCI-Expanded 1900-present，the Social Science Citation Index (SSCI 1900-present)，the Emerging Sources Citation Index (ESCI 2020-present).

The search by search formula #1 revealed no fully consistent bibliometric articles on mitochondria in myocardial ischemia in WOS. Based on the results of search formula #4, a total of 4,387 articles were retrieved in WOS and analyzed in the main text. (Supplementary Table 2, Supplementary Figure 1).

**Supplementary Table 2**

| **Rank** | **Search phrases** |
| --- | --- |
| #1 | TS=("Bibliometrics" OR "Bibliometric*" OR "Bibliometric Analysis" OR "Bibliographies, Statistical" OR "Statistical Bibliography" OR "Bibliography, Statistical" OR "Analysis, Bibliometric" OR "Analyses, Bibliometric" OR "Bibliometric Analyses" OR "Statistical Bibliographies" OR "Knowledge Mapping" OR "Knowledge graph" OR "Scientometric*" OR "Scientometrics" OR "Visualization" OR "CiteSpace" OR "VOSviewer") |
| #2 | TS=("Myocardial Ischemia" OR "Myocard* Ischemia*" OR "Cardiac* Ischemia*" OR "Ischemic Myocardi*" OR "Myocard* Infarction" OR "Myocard* Ischaemia*" OR "Ischemic Heart*" OR "Heart Ischemia*" OR "Ischemic Cardiomyopath*") |
| #3 | TS=("Mitochondria*" OR "Mitochondrion*") |
| #4 | #2 AND #3 AND LA=(English) AND DOP=(2015-01-01/2024-12-31) AND DT=(Article OR Review) |


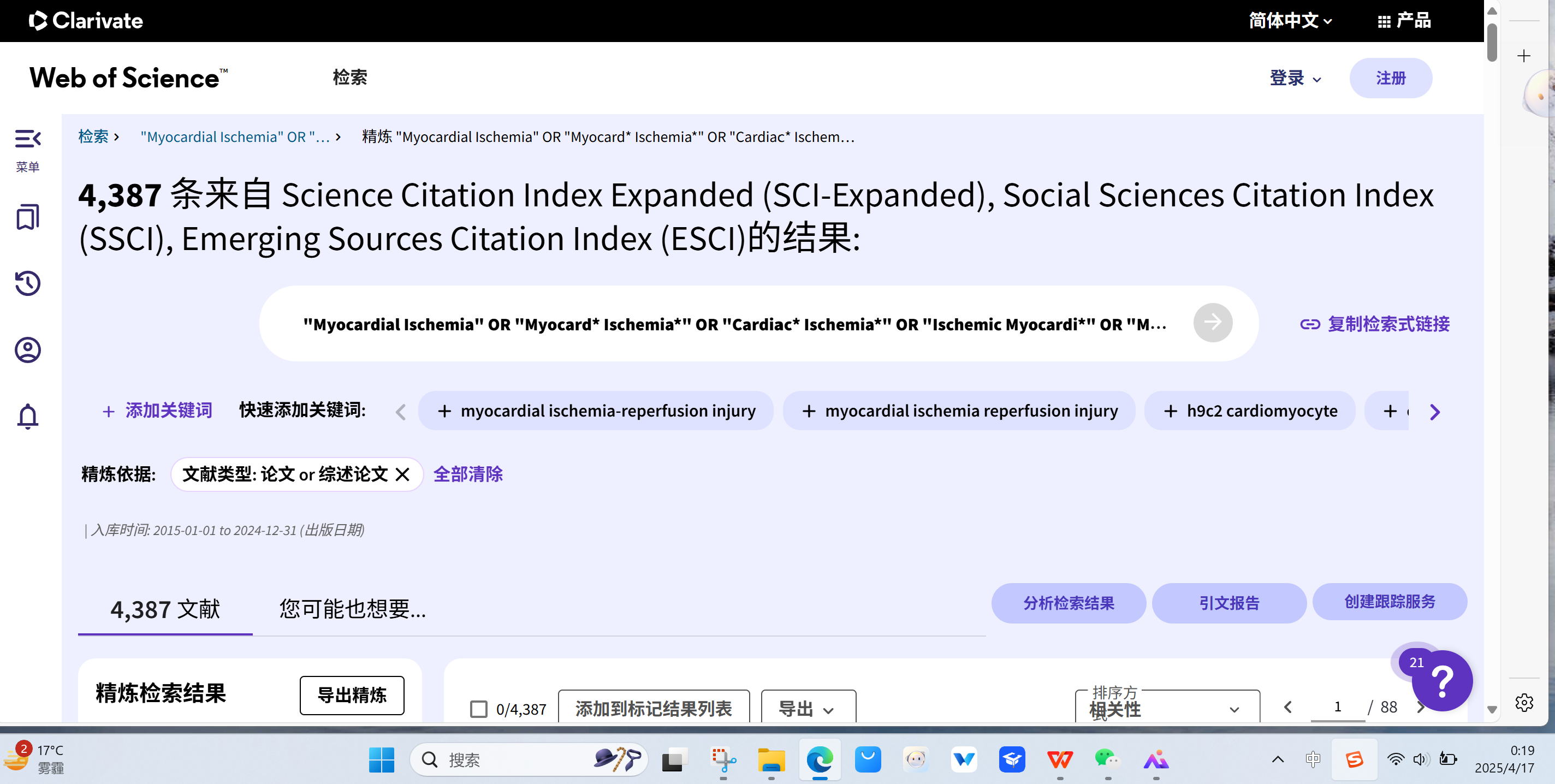


**Supplementary Figure 1.** Search results for Web of Science

**2.2 PubMed**

The search by search formula #1 revealed no fully consistent bibliometric articles on mitochondria in myocardial ischemia in PubMed. A total of 3,603 articles were retrieved from the pre-search of PubMed database by searching formula #4 (Supplementary Table 3, Supplementary Figure 2).

**Supplementary Table 3**

| **Rank** | **Search phrases** |
| --- | --- |
| #1 | ("Bibliometrics"[Mesh]) OR (("Bibliometrics" [Title/Abstract]) OR ("Bibliometric*" [Title/Abstract]) OR ("Bibliometric Analysis" [Title/Abstract]) OR ("Bibliographies, Statistical"[Title/Abstract]) OR ("Statistical Bibliography" [Title/Abstract]) OR ("Bibliography, Statistical"[Title/Abstract]) OR ("Analysis, Bibliometric" [Title/Abstract]) OR ("Analyses, Bibliometric" [Title/Abstract]) OR ("Bibliometric Analyses" [Title/Abstract]) OR ("Statistical Bibliographies" [Title/Abstract]) ("Knowledge Mapping" [Title/Abstract]) OR ("Knowledge Graph" [Title/Abstract]) OR ("Scientometric*" [Title/Abstract]) OR ("Scientometrics" [Title/Abstract]) OR ("Visualization" [Title/Abstract]) OR ("CiteSpace" [Title/Abstract]) OR ("VOSviewer" [Title/Abstract])) |
| #2 | ("Myocardial Ischemia"[Mesh]) OR ("Myocardial Ischemia"[Title/Abstract]) OR ("Myocard* Ischemia*"[Title/Abstract]) OR ("Cardiac* Ischemia*"[Title/Abstract]) OR ("Ischemic Myocardi*"[Title/Abstract]) OR ("Myocard* Infarction"[Title/Abstract]) OR ("Myocard* Ischaemia*"[Title/Abstract]) OR ("Ischemic Heart*"[Title/Abstract]) OR ("Heart Ischemia*"[Title/Abstract]) OR ("Ischemic Cardiomyopath*"[Title/Abstract]) |
| #3 | ("Mitochondria"[Mesh]) OR ((Mitochondria[Title/Abstract]) OR (Mitochondrion[Title/Abstract]) OR (Mitochondrial Contraction[Title/Abstract]) OR (Contraction, Mitochondrial[Title/Abstract]) OR (Contractions, Mitochondrial[Title/Abstract]) OR (Mitochondrial Contractions[Title/Abstract]) OR (Mitochondrial[Title/Abstract])) |
| #4 | #2 AND #3 AND (English[Language]) AND (2015/1/1:2024/12/31[pdat]) |


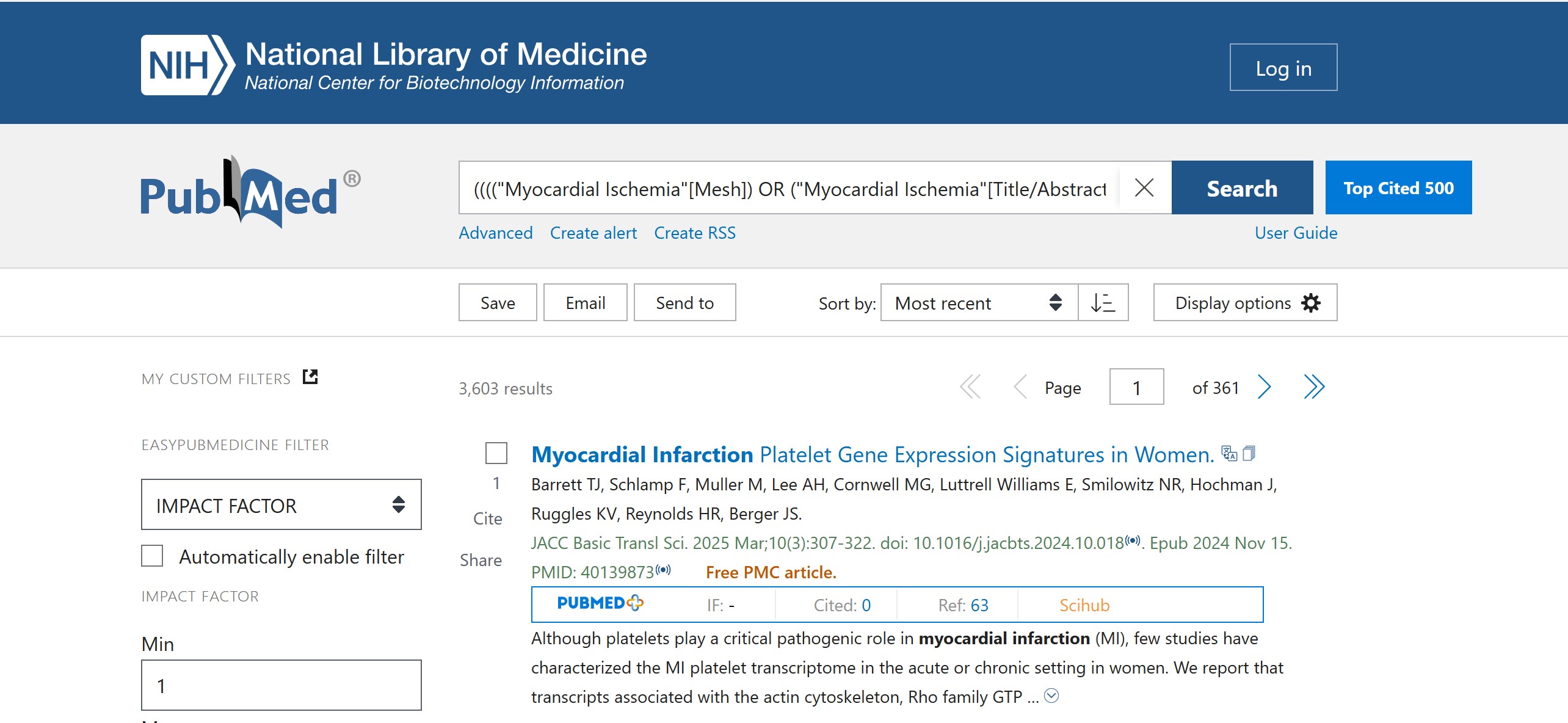


**Supplementary Figure 2.** Search results for PubMed
